# Navy Beans Impact the Stool Metabolome and Metabolic Pathways for Colon Health in Cancer Survivors

**DOI:** 10.3390/nu11010028

**Published:** 2018-12-22

**Authors:** Bridget A. Baxter, Renee C. Oppel, Elizabeth P. Ryan

**Affiliations:** Department of Environmental and Radiological Health Sciences, Colorado State University, Fort Collins, CO 80521, USA; bridget.baxter@colostate.edu (B.A.B.); renee.oppel@gmail.com (R.C.O.)

**Keywords:** colorectal cancer, dietary intervention, navy beans, metabolomics, stool, microbial metabolism

## Abstract

Colorectal cancer (CRC) is the third leading cause of cancer-related death in the United States and emerging evidence supports that increased consumption of legumes, such as navy beans, can reduce risk. Navy bean consumption was previously shown to modulate host and microbiome metabolism, and this investigation was performed to assess the impact on the human stool metabolome, which includes the presence of navy bean metabolites. This 4-week, randomized-controlled trial with overweight and obese CRC survivors involved consumption of 1 meal and 1 snack daily. The intervention contained 35 g of cooked navy bean or macronutrient matched meals and snacks with 0 g of navy beans for the control group (*n* = 18). There were 30 statistically significant metabolite differences in the stool of participants that consumed navy bean at day 28 compared to the participants’ baseline (*p* ≤ 0.05) and 26 significantly different metabolites when compared to the control group. Of the 560 total metabolites identified from the cooked navy beans, there were 237 possible navy bean-derived metabolites that were identified in the stool of participants consuming navy beans, such as *N*-methylpipecolate, 2-aminoadipate, piperidine, and vanillate. The microbial metabolism of amino acids and fatty acids were also identified in stool after 4 weeks of navy bean intake including cadaverine, hydantoin-5 propionic acid, 4-hydroxyphenylacetate, and caprylate. The stool relative abundance of ophthalmate increased 5.25-fold for navy bean consumers that can indicate glutathione regulation, and involving cancer control mechanisms such as detoxification of xenobiotics, antioxidant defense, proliferation, and apoptosis. Metabolic pathways involving lysine, and phytochemicals were also modulated by navy bean intake in CRC survivors. These metabolites and metabolic pathways represent an acute response to increased navy bean intake, which merit further investigation for improving colonic health after long-term consumption.

## 1. Introduction

Dry beans are staple foods and well recognized sources of vital macronutrients [1] and micronutrients such as folate, iron and magnesium [2]. Among the 17 grain legumes identified as food sources by the Food and Agriculture Organization of the United Nations, common bean (*Phaseolus vulgaris* L.) are among the most widely produced. There are economic, health and environmental arguments for increasing the consumption of common beans as a primary source of dietary protein and fiber in the diet. Additional attention is needed towards the functional properties of beans in Western diets that may reduce risk for major chronic diseases [3]. Overall common bean intake in the U.S.A was reported to be very low and approximately 58–65% of adults were below the recommended levels of intake [4,5,6,7,8].

Metabolomics was used as a tool in this study for identification of small molecules present in food and the metabolite changes conferred in the host after consumption. Metabolomics has helped to increase knowledge of the metabolic interactions between food components and gut bacteria that have products measurable in the human stool metabolome [9,10]. Dietary interventions in people have shown wide variations in metabolic responses that are often attributed to metabolism by varied gut microbiome structure and composition. There is limited information available for metabolite contents of specific foods that serve as substrates for gut commensal organisms, and with respect to the effects of food consumption on host metabolites following short versus long periods of time [11,12,13]. Integrating food and nutritional metabolomics are promising strategies that may lead to identification of candidate dietary biomarkers as the stool metabolome contains numerous food components including those transformed by host digestion and the gut microbiome.

An emerging body of research shows the chronic disease management and colon cancer prevention properties of legumes [14,15,16,17,18]. Common dry beans are comprised of phytochemicals and non-digestible fermentable components with anti-inflammatory and antioxidant properties [19,20], and common beans (i.e., navy, black, pinto and red) represent important components of diverse dietary patterns and demographics globally [2,21,22]. The polyp prevention trial demonstrated that increased levels of dry bean intake reduces the risk of advanced colorectal adenoma recurrence [23], and the National Institute of Health and Americans Association of Retired People (NIH-AARP) diet and health prospective study cohort showed that high fiber intake, particularly from legumes and beans, significantly lowers risk of all-cause mortality over a 9-year period [24,25]. Bean containing diets were also shown to exert beneficial effects during experimental colitis by reducing inflammatory biomarkers both locally and systemically [17]. Consumption of common beans has also been widely shown to lower total serum cholesterol levels in adults [26,27]. Notably, Borresen et al. [16,28] established that cooked navy bean powder was feasible to include in meals and snacks for increasing total dietary fiber intakes and that increased consumption is feasible at dose levels (5–10% of total dietary intake) associated with colon cancer protection in animals and humans [17,18,28,29]. Importantly increasing navy bean consumption was possible in adults and children without gastrointestinal difficulty [28,30].

Colorectal cancer is the third leading cause of death in the United States and there are opportunities to decrease the CRC risk through a healthy, legume-fiber rich diet and physically active lifestyle [25,31]. To bolster the study of gut health and chronic disease fighting properties of beans, dietary metabolite markers are needed in people. In this study, we evaluated the effect of navy bean consumption for 28 days and the impact on the stool metabolite profile. The stool metabolome includes a suite of metabolites that are endogenous from the host, products of the microbiome and exogenous from the diet and other environmental exposures, all of which can provide metabolic insights of high relevance to intestinal health [32]. We hypothesized that the stool metabolome would reveal candidate biomarkers of navy bean intake and identify host-microbe-food metabolite interactions that function as networks of metabolic pathways affected by increased navy bean consumption.

## 2. Materials and Methods

### 2.1. Study Design

A four-week, randomized-controlled, single-blinded dietary intervention trial recruited CRC survivors through the University of Colorado Health-North (UCH-North) Cancer Center Network. The study intervention was cooked navy beans (in a dried powder form) that were incorporated into meals and snacks. Each participant consumed 35 grams cooked navy bean powder/day, which is ~1/2 cup cooked whole navy beans. There were seven meals and six snacks developed by our team in partnership with the Colorado State University (CSU) Kendall Anderson Nutrition Center to cover a range of food preferences. The description of study provided foods herein that contained navy bean powder were previously reported [16,28]. The control group meals and snacks were nearly identical by macronutrient matching with the same food ingredients, but the meals and snacks for the controls did not include any cooked navy bean powder. The CONSORT diagram for the clinical trial is shown in Supplemental Figure S1 (NCT01929122 clinicaltrial.gov).

Eligibility criteria: participants were (1) more than four months post cancer treatment (e.g., chemotherapy or radiation), (2) body mass index (BMI) for overweight or obesity, (3) no history of other malignancies besides a CRC diagnosis, (4) no history of food allergies or major dietary restrictions, (5) not currently pregnant or lactating, (6) a non-smoker, (7) not taking antibiotics within the month prior to enrollment, and (8) no history of gallstones.

Randomization and study group allocation: participants who met eligibility criteria were randomized to control or navy bean intervention groups by BMI, sex, and daily caloric intake. Baseline characteristics of all participants are shown in Table 1. The Colorado State University Research Integrity and Compliance Review Board and the UCH-North Institutional Review Board approved this study protocol and informed consent (Protocol #s 09-1530H and 10-1038, respectively).

### 2.2. Cooked Navy Bean Powder for Dietary Intervention and Metabolomics

**Figure 1:**
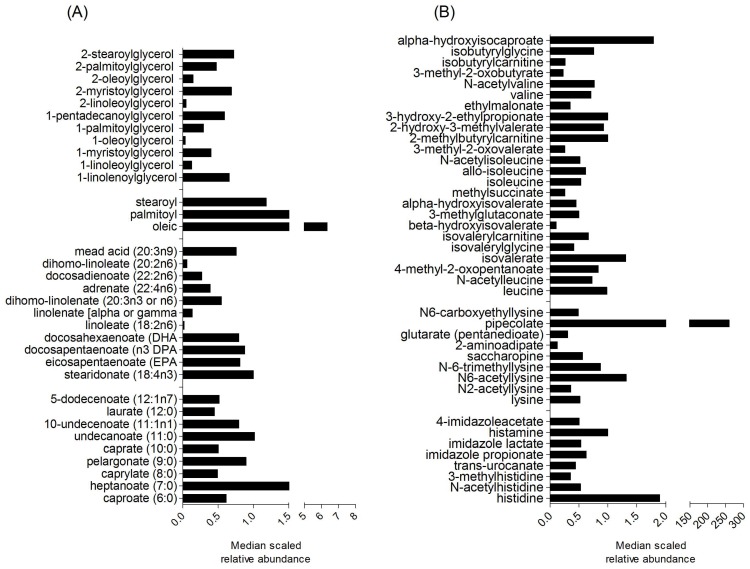
Median scaled relative abundances of selected navy bean metabolites classified as (**A**) lipids, and (**B**) amino acids. A complete list of the identified navy bean metabolites with the relative abundances can be found in Supplemental Table S1.

Archer Daniels Midland (ADM) Edible Bean Specialties, Inc. provided the Vegefull™ cooked navy bean powder (Decatur, IL, USA) that was incorporated into study meals and snacks as previously described [16,28,33,34]. For navy bean food metabolome determination, 100 mg of navy bean was subjected to non-targeted metabolite profiling at Metabolon (Durham, NC, USA). Navy bean was extracted with 80% MeOH and analyzed by gas-chromatography mass-spectrometry (GC-MS) and ultra-performance liquid chromatography mass-spectrometry (UPLC-MS/MS) in the positive and negative ionization mode platforms. The full list is provided in Supplemental Table S1 and selected metabolites are shown in Figure 1 from the navy bean metabolome.

### 2.3. Stool Sample Collection and Preparation for Metabolomics

Participants self-collected stool samples in specimen containers and all samples were transported to the Ryan laboratory at CSU within 24 hours of the study visit and immediately stored at −20 °C until further processing for metabolome analysis. Samples were collected at baseline (day 0) and day 28 (end of dietary intervention study). Stool samples were lyophilized, aliquoted and stored at −80 °C prior to metabolite extraction. Stool samples were extracted for metabolite profiling at Metabolon Inc. (Durham, NC, USA) [35]. Metabolites were extracted using 80% MeOH. The final extract was divided into 4 aliquots for metabolite analysis by different mass spectrometry applications described below.

### 2.4. Gas Chromatography-Mass Spectrometry (GC-MS) (Navy Bean and Stool)

Bistrimethyl-silyltrifluoroacetamide was used under nitrogen to derivative and separate samples on a 5% diphenyl/95% dimethyl polysiloxane fused silica column (20 m × 0.18 mm ID; 0.18 µm film thickness). The temperature ranged from 60 °C to 340 °C in a 17.5 minute period and helium was used as the carrier gas. Internal standards amyl benzene, 1-phenylhexane, 1- phenyloctane, 1-phenyldecane, 1-phenyldodecane, hexadecylbenzene, octadecylbenzene, tetradecylbenzene and 2, 6-di-tert-butyl-4-methylphenol were added to each sample (250 ng of each standard per sample). Samples were analyzed on a Thermo-Finnigan Trace DSQ fast-scanning single-quadrupole mass spectrometer using electro impact ionization (EI) and ran at unit mass resolving power; the scan range was from 50–750 *m*/*z*.

### 2.5. Ultra-Performance Liquid Chromatography-Mass Spectrometry (UPLC-MS/MS) (Navy Bean and Stool)

UPLC-MS/MS combines the physical separation capabilities of liquid chromatography with the mass analysis capabilities of mass spectrometry. The UPLC-MS/MS portion of the platform was based on a Waters ACQUITY UPLC and a Thermo-Finnigan LTQ MS operated at nominal mass resolution, which consisted of an electrospray ionization (ESI) source and linear ion-trap (LIT) mass analyzer. The dried sample extract was reconstituted in acidic or basic UPLC-compatible solvents, each of which contained 11 to 13 injection standards at fixed concentrations [36]. One aliquot was analyzed using acidic 10 positive ion-optimized conditions and the other using basic, negative ion-optimized conditions in two independent injections using separate dedicated columns (Waters UPLC BEH C18-2.1 × 100 mm, 1.7 µm). Extracts reconstituted in acidic conditions were gradient eluted using water and methanol containing 0.1% formic acid, while the basic extracts, which also used water/methanol, contained 6.5 mM ammonium bicarbonate. The MS analysis alternated between MS and data-dependent MS/MS scans using dynamic exclusion and the scan range was from 80–1000 m/z. Raw data was extracted, peak-identified and quality control (QC) processed as previously described [35,37].

### 2.6. Visualization of Metabolic Pathway Networks, Including Pathway Enrichment Score (PES)

**Figure 2:**
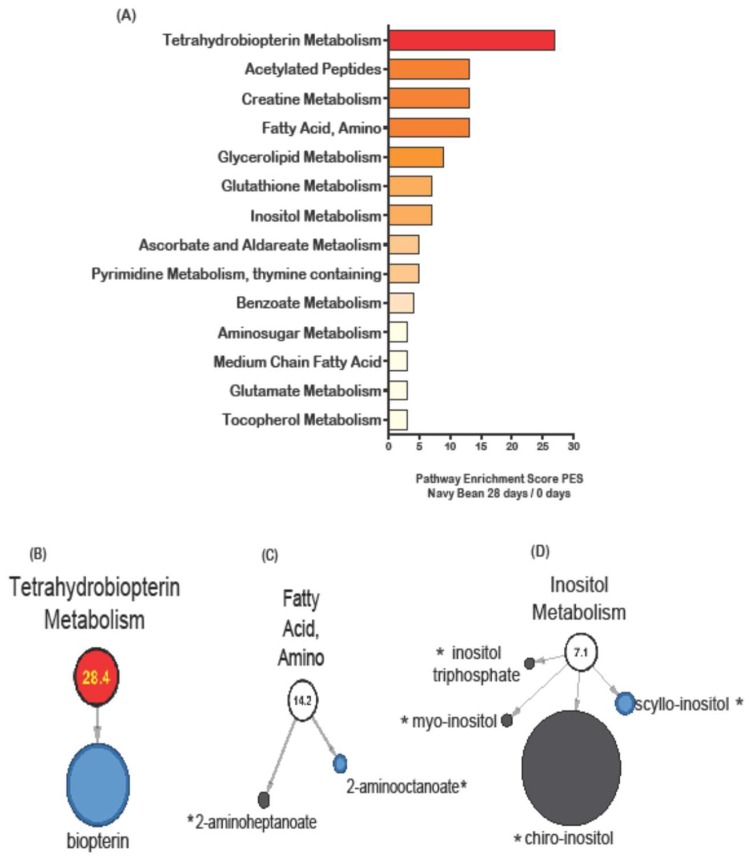
Stool metabolic pathways impacted by navy bean consumption as measured by pathway enrichment score (PES) for day 28 when compared to day 0 (baseline). (**A**) All stool metabolic pathways with a PES >2.0 are shown. Selected pathways with metabolites were shown for (**B**) tetrahydrobiopterin (1 metabolite) PES 28.4 (**C**) fatty acid, amino (2 metabolites) PES 14.2, and (**D**) inositol metabolism (4 metabolites), PES 7.1. Metabolites with black node were not significantly changed by beans. Red nodes indicate significantly increased metabolite abundance and blue node represents significantly decreased metabolite after consuming navy beans for 28 days compared to baseline (day 0). The size of the circle reflects the relative abundance scaled to all stool metabolites. The * indicates presence in the navy bean metabolome.

**Figure 3:**
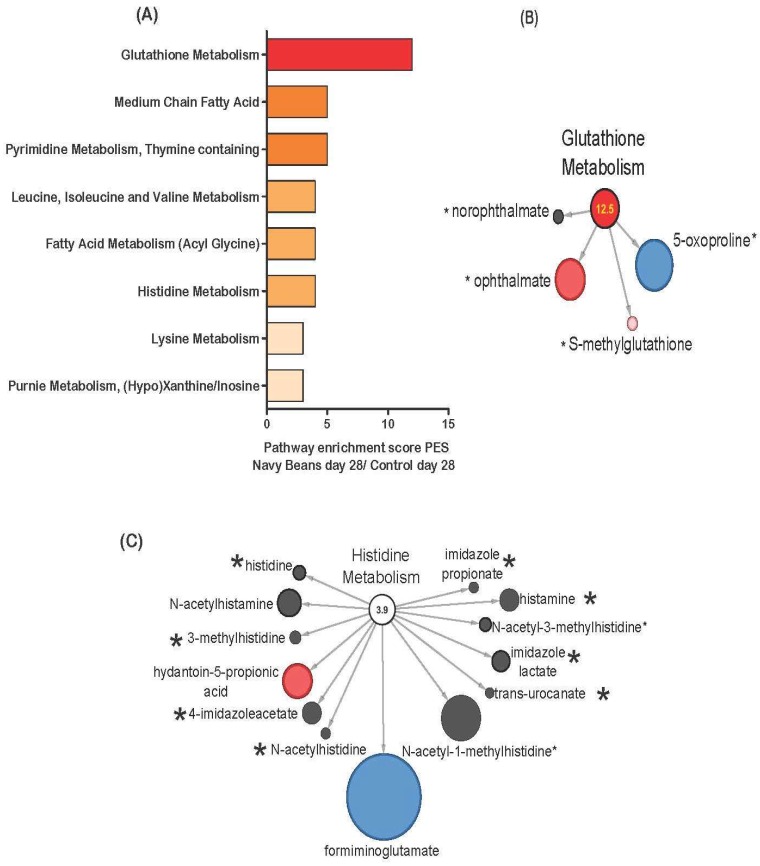
Stool metabolic pathways impacted by navy beans consumption as measured by pathway enrichment score (PES) and compared to control at day 28. (**A**) All metabolic pathways with a PES > 2.0, (**B**) glutathione metabolism (4 identified navy bean metabolites) PES 12.5, and (**C**) histidine metabolism (8 identified navy bean metabolites) PES 3.9. Red nodes indicate significant increase, blue node is significant decrease, and light pink shows trending increase in abundance between navy beans and control group at 28 days. The size of the circle reflects the relative abundance when scaled to all stool metabolites. The * indicates presence in the navy bean metabolome listed in Supplementary Table S1.

To visualize the metabolic pathways for the suite of stool metabolites identified, the relative abundance of each metabolite for each metabolic pathway were loaded into a pathway analysis software and metabolite classification system called Metabolync (portal.metabolon.com). Pathway enrichment scores (PES) were calculated by dividing the number of significant compounds in a pathway by all metabolites identified in that pathway, and then again divided by the total number of significant metabolites in the entire dataset over total metabolites detected [37]. Pathways with a PES >2 were selected for display as these metabolic pathways contained metabolites with significant changes in the stool relative abundance after one month (Figure 2) or significant differences in abundance between groups at day 28 (Figure 3). A cut off >2 were applied herein to focus on metabolic pathways with measurable stool metabolite responses to increased navy bean consumption. Metabolic pathways with a PES > 10 were described in greater detail for modulation by navy bean consumption over the 28 day period (Figure 2b–d) and between control and navy bean groups at day 28 (Figure 3b,c). Metabolites were visualized within a metabolic pathway via Cytoscape v 2.8.3 (Institute of Systems Biology, Seattle, WA, USA). Each metabolite is shown with a black, blue or red colored node extending from a sub-metabolic pathway node, which then connects to a super-metabolic pathway hexagonal node. A black node represents metabolite identified. A red node represents metabolites with significantly (*p* ≤ 0.05) higher expression at day 28 compared to baseline. A blue node represents metabolites with significantly lower expression at day 28 compared to baseline. Node size is proportional to median scaled relative abundance of the metabolite.

### 2.7. Statistical Analyses

Stool metabolite profiles were quantified by the metabolite relative abundance and median scaled to 1. Statistical analysis was completed between control group and navy bean intervention treatment group at day 28, and overtime (day 0 to day 28). *p*-values of ≤0.05 were considered statistically significant. A matched-pairs 2-way analysis of variance (ANOVA) with post-test contrasts was completed using the scaled relative abundance of each metabolite in order to evaluate the impact of dietary navy bean supplementation over 4 weeks in the stool metabolome. An estimate of false discovery rate (*q*-values) was calculated to determine false discoveries common to multiple-comparison metabolomics studies. Metabolites with *q*-values ≥ 0.1 were excluded from further analysis. Only the known compound identities from navy bean metabolome were used for comparison with overlap to stool metabolites.

## 3. Results

### 3.1. Navy Bean (Food) Metabolome

There were 560 metabolites with confirmed identities from the methanol extracted cooked navy bean powder. Relative abundance of all navy bean metabolites are listed in Supplemental Table S1. The 560 identified navy bean metabolites were classified broadly across eight chemical classes; amino acids, carbohydrates, cofactors and vitamins, energy, lipids, nucleotides and phytochemicals. Figure 1 shows visual representation of selected navy bean, lipids, and amino acids. There are 164 lipids identified from the navy bean metabolome that include 30 sub-metabolic pathways. Figure 1A shows monoacylglycerol, endocannabinoid, polyunsaturated fatty acid (*n*3 and *n*6), and medium chain fatty acids. Out of the 144 identified navy bean amino acids, Figure 1B shows the metabolites derived from leucine, isoleucine and valine, histidine, and lysine metabolic pathways. There are also 31 cofactors and vitamins, 43 carbohydrates, 11 energy metabolites, 54 nucleotides, 31 peptides and 82 other phytochemicals (see complete list in Supplemental Table S1). The navy bean metabolite profile was next assessed for its presence and overlap with the stool metabolome of CRC survivors after one month of dietary navy bean intake.

### 3.2. Dietary Modulation of Stool Metabolite Composition with One Month Exposure

Stool metabolites were classified into eight chemical classes (i.e., amino acids, carbohydrates, lipids, energy, peptides, cofactors and vitamins, nucleotides, food components/plant) that are organized into metabolic pathway networks, and analyzed by diet group. After 28 days of navy bean consumption in study meals and snacks, there were a total of 6 chemical classes (15 amino acids, 6 carbohydrates, 19 lipids, 3 nucleotides, 3 cofactors and vitamins and 21 phytochemicals) with significant increases or decreases in stool metabolite relative abundance when compared to baseline. Stool metabolites for both groups are presented as the mean fold change over time, and with respect to the scaled relative abundance of each metabolite for all participants. Table 2 shows 67 stool metabolites that significantly changed and 31 metabolites significantly changed after CRC survivors consumed 35g of cooked navy bean incorporated into meals and snacks daily for 28 days. For the navy bean group, 12 of the 31 metabolites increased and 19 metabolites decreased (*p*-value ≤ 0.05). Stool metabolites from Table 2 that increased after cooked navy bean consumption are anacardic acid (9.38 fold change), 4-hydroxyphenylacetate (4.51 fold change), cadaverine (3.20- fold change), diacetylchitobiose (3.42 fold change), and 5,6 dihydrothymine (1.69 fold change). There are 5 metabolites with significant increases that were also identified as navy bean metabolites; ophthalmate (5.25 fold change), piperidine (2.59 fold change), adenosine-2′, 3′-cyclic monophosphate (1.31 fold change), and *N*-methylpipecolate (1.57 fold change). Stool metabolites such as phenylacetylglglutamine (0.09 fold change), 2-(4-hyroxyphenyl) propionate (0.03 fold change), and glycocholenate sulfate (0.08 fold change) may also be derived from navy beans, and were shown to have lower abundance in stool over time. Additional metabolites that had a significant decrease in the navy bean consumers after day 28 that were also identified as navy bean metabolites included guanidinoacetate (0.07 fold change), caprylate (0.31 fold change), glutamate (0.82 fold change), creatine (0.10 fold change), *N*-acetylmethioninie sulfoxide (0.43 fold change), scyllo-inositol (0.52 fold change), and 2-aminooctanoate (0.68 fold change). A decrease in relative abundance of a navy bean metabolite may indicate uptake into the colon tissue and bioavailability to the host and, therefore, be measured as a lower amount excreted stool. From the 31 metabolites that were significantly modulated after one month of consuming navy beans, 14 metabolites were dually detected in the stool and navy bean metabolome. Also noteworthy from this analysis are the 40 distinct stool metabolites that significantly differed in CRC survivors consuming a control diet (0 g beans/day). Of the 40 metabolites with significant fold changes over time in the control group, 30 metabolites increased and 10 metabolites decreased in abundance. Stool metabolites that changed in control group over the 28 day period were considered a response to the study provided meals and snacks without beans. The metabolites affected by control foods were *N*-acetylasparagine (2.37 fold change), docosatrienoate (3.96 fold change), 3-hydroxysebacate (3.20 fold change), 3-hydroxybenzoate (3.20 fold change), 1-methylurate (0.74 fold change), and 7-methylurate (0.94 fold change). Stool metabolites that increased or decreased in both control and navy bean groups were enterolactone, increased in navy bean (2.81 fold change) and control (2.00 fold change), 3-(2-hydroxyphenyl)propionate increased navy bean (2.74 fold change) and control (2.99 fold change) and sitostanol decreased in both groups, (navy bean 0.79 fold change) and (control 0.59 fold change). Also one stool metabolite 1-myristoylglycerol (1-monomyristin) decreased in the navy bean consumers group (0.29 fold change) and increased in the control group (2.54 fold change).

### 3.3. Stool Metabolic Pathways Impacted by Control and Navy Bean Groups after One Month

The metabolic pathway enrichment score (PES) was calculated for all stool metabolites in each study diet group. PES were calculated by dividing the number of significant metabolites in a single metabolic pathway by the total number of metabolites identified in that pathway and the entire metabolome. Figure 2A shows the metabolic PES on day 28 compared to baseline for the navy bean intervention group. The amino acid metabolic pathways affected by navy bean consumption were phenylalanine and tyrosine metabolism (acetylated peptides) (14.2 PES), creatine metabolism (14.2 PES), glutathione metabolism (7.1 PES), and glutamate metabolism (2.69 PES). The lipid metabolic pathways were fatty acid, amino (14.2 PES), glycerolipid metabolism (9.0 PES), inositol metabolism (7.1 PES) and medium chain fatty acids (2.99 PES). Cofactors and vitamins were affected after 4 weeks of navy bean consumption, and included tetrahydrobiopterin metabolism (28.4 PES), ascorbate and aldareate metabolism (5.39 PES), and tocopherol metabolism (2.69 PES). The phytochemicals with the highest PES was benzoate metabolism (4.14 PES), for carbohydrates was amino sugar metabolism (2.99 PES), and for nucleotides was pyrimidine (thymine containing) (5.39 PES). Figure 2B shows that the significant decrease in the biopterin metabolite abundance was driving the elevated enrichment score for the tetrahydrobiopterin metabolism pathway. Biopterin was identified in the cooked navy beans and was significantly decreased in the stool. A decreased abundance in stool may suggest uptake into the colon. Figure 2C shows the fatty acid amino metabolism pathway with two metabolites (2-aminoheptanoate and 2-aminooctanoate) that are dually identified in the cooked navy bean and stool metabolome. Figure 2D shows inositol metabolism impacted by four metabolites in the stool metabolome, including inositol triphosphate, myo-inositol, chiro-inositol and scyllo-inositol (all of these are potential bean-derived metabolites with scyllo-inositol significant for decreased abundance in stools).

### 3.4. Stool Metabolite Distinctions between Navy Bean and Control Group at End of Study

After 28 days of cooked navy bean consumption in study foods, there were multiple chemical classes impacted when compared to the control group at day 28, namely 10 amino acids, 1 peptide, 1 energy, 7 lipids, 4 nucleotides, and 3 phytochemicals. The entire list of stool metabolites identified from all participants is provided in Supplementary Table S2. Table S3 shows the 26 stool metabolites that had a significant fold difference between the control group and the navy bean intervention group at day 28. Stool metabolites are presented as the mean fold difference between the groups. From the 26 metabolites that showed an increased or decreased fold difference after day 28, 18 of them are dually detected in the stool metabolome and navy bean metabolome. Of the 26 metabolites with significant fold differences in the navy bean group, 8 metabolites increased with a fold difference greater than 1, and 18 metabolites decreased that had a fold difference less than 1. Stool metabolites from Table 3 selected for colon health are *N*2,*N*6-diacetyllysine (3.17 fold difference), and nobiletin (1.48 fold difference). The significantly increased stool metabolites that were also identified as navy bean metabolites were ophthalmate (3.49 fold difference), 2-aminoadipate (2.69 fold difference), gamma-glutamylglutamine (2.60 fold difference), and guanosine 5′- monophosphate (5′-GMP) (1.07 fold difference). Selected stool metabolites with significant decreases include formiminoglutamate (0.11 fold difference), 3-methyl-2-oxobutyrate (0.41 fold difference), and glycocholenate sulfate (0.10 fold difference). Stool metabolites that significantly decreased were also identified as navy bean metabolites such as, *N*-acetylisoleucine (0.15 fold difference), valerylglycine (0.13 fold difference), 4-methyl-2-oxopentanoate (0.41 fold difference), 3-methyl-2-oxovalerate (0.37 fold difference), malate (0.39 fold difference), 1-myristoylglycerol (0.31 fold difference), 5-oxoproline (0.23 fold difference), vanillin (0.51 fold difference), undecanoate (0.50 fold difference), allantoin (0.11 fold difference) and caprylate (0.1 fold difference), salicylate (0.77 fold difference), vanillin (0.51 fold difference).

### 3.5. Network of Metabolic Pathway Differences Between Control and Navy Bean Groups after the One Month Feeding Period

Figure 3A shows the stool metabolic pathways impacted by navy bean consumption as measured by PES and with comparison to control intake at day 28. The amino acid metabolic pathways affected by navy bean consumption at day 28 when compared to the control group at day 28 were glutathione metabolism (12.5 PES), leucine, isoleucine and valine metabolism (4.5 PES), histidine metabolism (3.9 PES), and lysine metabolism (2.97 PES). The lipids metabolic pathways that differed from control were medium chain fatty acid (5.24 PES), and fatty acid metabolism (acyl glycine) (4.2 PES). Nucleotide metabolism after 4 weeks of navy bean consumption differed for pyrimidine metabolism, (thymine containing) (4.75 PES), purine metabolism, and (hypo) xanthine/inosine (2.97 PES). Visual representation for the dual identification of metabolites between the cooked navy bean metabolome and the stool metabolome, are shown in Figure 3B-C. Figure 3B shows the four metabolites from the glutathione metabolism pathway in the navy bean stool metabolome when compared to the control stool metabolome at day 28 that were also in the cooked navy bean metabolome, including norophthalmate, ophthalmate, S-methylglutathione, and 5-oxoproline. Figure 3C shows 13 histidine metabolites from the stool metabolome, 8 of which are dually identified in the cooked navy bean metabolome (i.e., histidine, 3-methylhistidine, 4-imidazoleacetate, histamine, imidazole lactate, trans-urocanate, and imidazole propionate).

### 3.6. Overlap between Navy Bean (Food) Metabolome and Stool Metabolome

**Figure 4:**
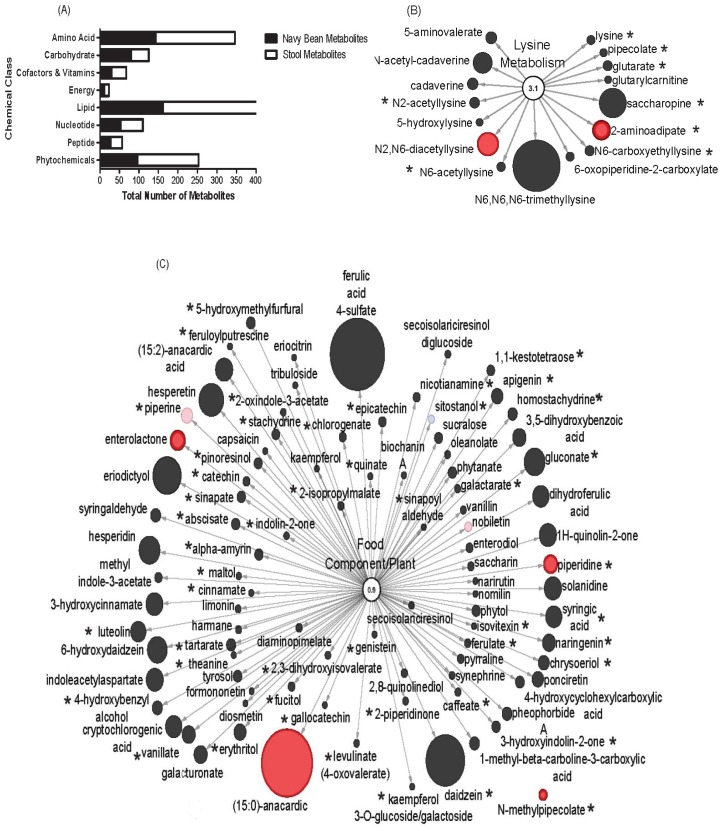
Overlap between navy bean (food) metabolome and stool metabolome of navy bean consumers. (**A**) The stool metabolome contained 782 components and the overlap of metabolites from the cooked navy bean food (367 metabolites). Pathways of (**B**) lysine metabolism (16 metabolites) and (**C**) food and plant components (96 metabolites). Stool metabolites with black were not significant in the navy bean group over baseline. Red nodes indicate significant increase after consuming navy beans when compared to the baseline. The size of the circle relates to the relative abundance when scaled to all stool metabolites. The * indicates presence in the navy beans.

The stool metabolome from navy bean consumers were next examined for navy bean-derived components. There were 560 named biochemicals from the cooked navy bean food metabolome (Supplementary Table S1). The stool metabolome of the navy bean consumers was comprised of 782 named biochemicals that are provided in Supplementary Table S2. The 367 metabolites that have overlap between the food and stool were determined by having an identical name or were a related metabolite identity following biotransformation. Figure 4A shows the 367 metabolites that overlap between navy bean metabolome and stool metabolome that were classified as 96 amino acids, 121 lipids, 20 cofactors and vitamins, 10 energy metabolites, 24 nucleotides, 51 phytochemicals, 29 carbohydrates and 16 peptides. In this study, it is not possible to confirm that the metabolite origin from the navy beans is the same metabolite that was identified in stools. This analysis merely identified overlapping metabolites from navy beans (food metabolome) and the stool (nutritional metabolome response). Amino acids, lipids and phytochemicals represent the largest cluster of identified metabolites with overlap in stool and navy bean food metabolomes. Figure 4B shows lysine metabolism impacted by navy beans with 16 metabolites in the pathway, and 8 metabolites are navy bean-derived (i.e., *N*2-acetyllysine, *N*6-acetyllysine, lysine, pipecolate, glutarate, saccharopine, 2-aminoadipate and *N*6-carboxyethyllysine). Figure 4C shows numerous food and plant metabolites from stools, and highlights the 47 of these that are also in the navy bean metabolome, such as *N*-methylpipecolate, piperine, piperidine, sitostanol, cinnamate, stachydrine, nicotianamine and syringic acid.

## 4. Discussion

Increasing fiber intake after a CRC diagnosis was recently associated with lower CRC specific and overall mortality [38]. Beans represent a promising food for achieving the recommended increase in fiber, but also deliver multiple other bioactive components with benefits to the colon. Using a non-targeted metabolomics approach, we demonstrated that navy bean consumption for 4 weeks modulated the stool metabolome of CRC survivors across multiple metabolic pathways of relevance to improving colon health and reducing CRC risk. This study revealed stool metabolites that were also indicative of compliance to navy bean consumption as measured following host and/or gut microbial metabolism. Navy bean consumption modulated major metabolic pathways such as sterol, lysine, fatty acid, amino and inositol metabolism that were previously shown to be protective against CRC [39,40,41,42]. Dietary navy bean intake (35 g/day) increased the stool relative abundance of several amino acids, bean derived phytochemicals, and lipids. The impact of navy bean consumption on changes in the stool metabolite profile may be dose related for colonic health importance, as the study participants had 8–10% of total calories coming from navy beans in the diet at day 28 [28]. Detection of navy bean metabolites does not clearly indicate which gut microbial communities were active players in enzymatic biotransformation, yet these microbial modified, bean-derived metabolites merit attention for having novel mechanisms of action in the gut directly or indirectly to reduce CRC.

The sterols, glutathione metabolites, lysine components, as well as other bean phytochemicals merit continued investigation for colon cancer control and prevention following different doses and durations of intake. Four sterol metabolites, namely lanosterol, beta-sitosterol, campersterol and fucosterol were all trending towards increased abundance in stools after navy beans intake, yet narrowly missed statistical cutoff for significance 0.05 < *p* < 0.10 due to extensive inter-individual variations at baseline, yet these are notably identified as navy bean metabolites. The sterols comprise an example metabolic pathway for assessment in future studies for consideration at baseline (i.e., time of randomization) to reduce baseline variability between groups. Phytosterols, plant sterols and stanols, originate from the diet and are also present in nuts, vegetable oils, seeds, cereals and beans [43]. These compounds are structurally similar to cholesterol and characterized by anti-carcinogenic and anti-antherogenic properties [44]. The potential mechanisms of the protective effect of navy bean-derived phytosterols against CRC include inhibition of the cell cycle progression, induction of cellular apoptosis and the reduction of cellular oxidative stress [45]. β-sitosterol is a component of beans that has been shown to inhibit growth of COLO 320 DM cells (IC50 266.2 μM), by scavenging reactive oxygen species and inducing apoptosis; as well as suppressing the expression of β-catenin and PCNA antigens in human colon cancer cells [21,22]. Additionally, Baskar et al. [46] showed that β-sitosterol supplementation reduced the number of aberrant crypt and crypt multiplicity in the 1,2-dimethydrazine (DMH)-initiated rats in a dose-dependent manner with no toxic effects . Dietary administration of lanosterol has the potential to suppress chemically induced carcinogenesis in a model assay [47]. Fucosterol has also shown to have antidiabetic, anti-inflammatory, and antioxidant properties in addition to anticancer effects [48]. An inverse association was previously shown between the consumption of β-sitosterol, campesterol, and camperstanol and cancer risk [24,25].

Beans contain protein that was hypothesized herein to deliver glutathione and lysine, which modulate essential amino acid and redox metabolism. The protein contribution of navy beans to the diet is of particular importance as many amino acid metabolite contents in stools were increased. In addition, digestibility of the dry bean protein was previously reported to be 67–73% [49]. Glutathione synthesis and nutritional status are critical to the development of effective therapeutic strategies to prevent and treat a wide array of human diseases, including cancer [50]. Glutathione metabolism significantly increased when compared to control at 28 days and had a PES of 11.2. Pyroglutamic acid, also known as 5-oxoproline is an intermediate in the glutathione pathway and identified as a navy bean metabolite which decreased after 28 days of navy bean consumption compared to the control. Ophthalmate, a glutathione metabolite that increased in the stool of navy bean consumers was identified as an indicator of hepatic glutathione (GSH) depletion in the blood, and may be a biomarker for oxidative stress [51]. Lysine, an essential amino acid, plays an important role in cell proliferation and metabolism and is found in navy beans. Long-term lysine restriction from piglets to finishing pigs affected overall amino acid metabolism, which might be associated with gut microbiota and the 5’ adenosine monophosphate-activated protein kinase (AMPK) signaling pathway [52]. Cadaverine is another lysine metabolite that supports the significant upregulation of protein metabolism, and was previously associated with increased bean consumption [8]. In this study when comparing navy bean to control group at day 28, 2-aminoadipate (lysine metabolite) significantly increased (2.69 fold difference) in stool and was identified in navy beans. In previous studies, 2-aminoadipate was highlighted in Brown et al. [37] showed significance between colorectal cancer tissue from adjacent mucosa, and Zhu et al. [53] reported significant decrease in serum of colorectal cancer patients’ vs. healthy adults. The reported 2-aminoadipate higher in CRC disease when compared to the stable disease suggests that increased excretion in stool with navy beans may be protective [54].

Eicosapentaenoic acid (20:5*n*3) and scyllo-inositol are two metabolites that decreased in stool after day 28 of navy bean consumption compared to baseline and both were identified as navy bean metabolites. The lower stool abundance of eicosapentaenoic acid (20:5*n*3), a polyunsaturated fatty acid, may be a result of enhanced colonic uptake after bean consumption by the host. Eicosapentaenoic acid may exert anticancer actions by influencing various stages of cancer progression, including cell proliferation, cell survival, angiogenesis, inflammation and metastasis [55,56]. Scyllo-inositol, a product of inositol metabolism, demonstrated cancer preventive effects both in vitro and in vivo [41].

There were also three metabolites from the cofactors and vitamins that decreased in stools after navy bean consumption compared to baseline and were identify as navy bean metabolites. Ascorbate has been shown to exert anti-tumor activity [57], and gamma-CEHC showed anti-inflammatory activities in vivo that may be important for human disease prevention and therapy [58]. Biopterin derived from tetrahydrobiopterin metabolism had the highest PES in the navy bean intervention group when compared to baseline and, therefore, this navy bean-derived metabolite merits continued investigation in long-term feeding studies. Depleting biopterin levels in the tumor microenvironment could inhibit tumor angiogenesis at the same time increasing production of cytotoxic superoxide [59].

Polyphenols are phytochemicals that are abundant in many plant foods, including beans, and deliver broad-spectrum antioxidant properties. The primary functions of polyphenols as anti-oxidants were involved in the prevention of degenerative diseases such as cancer and metabolic syndromes [60,61]. The anti-carcinogenic and anti-mutagenic activities of common dry beans are strongly associated with the presence of phenolic compounds as well as other bioactive components; Bennink et al. [62] investigated diets fed with black bean and navy beans in rats and observed decreased occurrence of total tumors (59%) and adenocarcinomas (44%) [63]. Two phytochemicals (anacardic acid and nobiletin) with antitumor and antimicrobial activity were notably increased in stool after navy bean intake and were not identified in navy beans [61,64].

Piperidine, enterolactone, *N*-methylpipecolate, and salicylate are other phytochemicals from navy beans that increased in stool after one month of navy bean intake and have been shown to have antitumor activity or cancer preventive properties. Piperidine was shown to inhibit the proliferation of several breast cancer cell lines [31] and inhibit growth of colon cancer cells [65], *N*-methylpipecolate has been shown, alongside lowering cholesterol, to be relevant for cardiovascular disease risk reduction in adulthood [33]. Enterolactone is a product of the gut microbiome and lipid metabolism that influences fatty acid transporters [66] and was reported to have an antitumor effect [67]. Higher intake of fiber along with high urinary enterodiol concentration may have positive health benefits [68]. Salicylate was identified as a navy bean metabolite that decreased in navy bean consumers at 4 weeks when compared to baseline. Salicylate occurs naturally in many plants and has numerous evidences for being highly effective for treating colon cancer patients post diagnosis [69]. While most of the evidence stems from studies with aspirin, the utility of food-derived salicylate for colon cancer prevention warrants attention.

## 5. Conclusions

Beans contain a number of components, such as 2-aminoadipate, ophthalmate, eicosapentaenoic acid, scyllo-inositol, gamma-glutamylglutamine, *N*-methylpipecolate, and piperidine that have been implicated in protection against colon cancer. This randomized controlled intervention study in a cohort of individuals at high risk for CRC recurrence provides the first stage of evidence for the response to navy bean consumption as measured in stools. This study also included a profile of navy bean food metabolites that were assessed in stool after increased levels of intake. The next stage of validation of dietary biomarkers for navy beans should involve targeted metabolite assays. These bean-derived metabolites in stools can be used as compliance dietary biomarkers of navy bean consumption and/or as biomarkers for colon health. Identification of an overlap in the navy bean metabolome with the stool metabolome confirmed the possible role for several amino acids, lipids and phytochemicals to undergo host and gut microbial metabolism. Nutritional metabolomics approaches warrant continued application in larger cohort investigations with common dry beans, and should be of longer duration (i.e., 3–12 months), different doses or levels of intake (0–25% calories from the daily diet) and with distinct types of common beans (e.g., black, pinto, red kidney). Evaluating dietary exposure markers of beans in diverse populations will further advance the utility of these findings and could assist in the establishment of public health recommendations for levels of legume intake needed for colon cancer control and prevention.

## Figures and Tables

**Table 1:** Baseline characteristics of study population.

	Control (*n* = 10)	Navy Bean (*n* = 8)
Age (years)	65.50 ± 3.07	60.9 ± 11.0
Sex	4 (40%)	2 (22%)
Males (%) Females (%)	6 (60%)	7 (78%)
BMI (kg/m^2^)	27.26 ± 3.29	25.9 ± 5.0
Fiber Intake	25.07 ± 11.2	19.94 ± 10.0

Values are presented as mean ± SD.

**Table 2:** Stool metabolites that change over time from participants consuming control or navy bean intervention meals and snacks.

Metabolic Pathway	Biochemical Name	Control (day 28/day 0)		Navy Beans (day 28/day 0)	
		Fold Change	*p*-Value	Fold Change	*p*-Value
**Amino Acid**					
Alanine and Aspartate	*N*-acetylasparagine	**2.37**	0.0498	0.88	0.8519
	*N*-propionylalanine	**1.05**	0.0062	0.74	0.8716
Glutamate	glutamate	1.14	0.2647	**0.82**	0.0372
Lysine	2-aminoadipate	**0.74**	0.0331	1.01	0.5629
	cadaverine	1.20	0.961	**3.20**	0.0009
Phenylalanine and Tyrosine	4-hydroxyphenylacetate	1.03	0.7962	**4.51**	0.0181
	3-hydroxyphenylacetate	**2.43**	0.0020	0.77	0.7575
	phenylacetylglutamine	1.68	0.8156	**0.09**	0.0494
	2-(4-hydroxyphenyl)propionate	1.22	0.2846	**0.03**	0.0110
Tryptophan	tryptamine	**1.20**	0.0032	0.29	0.1752
Methionine, Cysteine, S-adenosylmethione (SAM) and Taurine	*N*-acetylmethionine sulfoxide	1.17	0.6650	**0.43**	0.0428
	homocysteine	**1.67**	0.003	1.11	0.5865
Creatine	creatine	0.78	0.9114	**0.10**	0.0074
	guanidinoacetate	3.21	0.4745	**0.07**	0.0497
Glutathione	ophthalmate	1.13	0.8969	**5.25**	0.0116
**Carbohydrate**					
Pentose	ribose	**1.38**	0.0261	0.92	0.7411
	ribonate	**22.89**	0.0159	0.91	0.9095
	xylonate	**12.63**	0.0029	1.57	0.3878
	2-deoxyribose	1.42	0.1133	**1.62**	0.0269
Aminosugar	diacetylchitobiose	1.89	0.6057	**3.42**	0.0066
Advanced Glycation	*N*6-carboxymethyllysine	**1.14**	0.0076	0.95	0.6244
**Lipid**					
Medium Chain Fatty Acid	caprylate (8:0)	3.94	0.1863	**0.31**	0.0441
	laurate (12:0)	**8.15**	0.0175	0.14	0.4344
Long Chain Fatty Acid	myristate (14:0)	**3.43**	0.0186	0.37	0.4247
	cis-vaccenate (18:1*n*7)	**1.99**	0.0335	0.94	0.7617
Polyunsaturated Fatty Acid (*n*3 and *n*6)	docosahexaenoate (DHA; 22:6*n*3)	**21.69**	0.0099	1.13	0.6900
	docosatrienoate (22:3*n*3)	**3.96**	0.0094	1.36	0.8702
	linoleate (18:2*n*6)	**1.58**	0.0499	0.84	0.6734
	arachidonate (20:4*n*6)	**9.64**	0.0128	1.05	0.9502
	docosadienoate (22:2*n*6)	**4.83**	0.0414	1.79	0.8219
Fatty Acid, Amino	2-aminooctanoate	0.99	0.154	**0.68**	0.0285
Fatty Acid, Monohydroxy	3-hydroxysebacate	**3.20**	0.0242	0.54	0.7037
Inositol	scyllo-inositol	0.74	0.2135	**0.52**	0.0473
Glycerolipid	glycerol 3-phosphate (G3P)	1.00	1.0000	**0.63**	0.0454
Monoacylglycerol	1-myristoylglycerol (1-monomyristin)	**2.54**	0.0384	**0.29**	0.0132
Sphingolipid	*N*-acetylsphingosine	**1.87**	0.0159	0.50	0.4079
Steroid	5alpha-androstan-3alpha,17beta-diol disulfate	**3.24**	0.0453	0.63	0.5735
Secondary Bile Acid	7,12-diketolithocholate	**0.24**	0.0251	23.33	0.5602
	6-oxolithocholate	1.02	0.9860	**0.49**	0.0210
	glycocholenate sulfate *	1.10	0.3844	**0.08**	0.0118
**Nucleotide**					
Purine, (Hypo)Xanthine/Inosine containing	hypoxanthine	**1.91**	0.0258	1.46	0.8596
Purine, Adenine containing	adenosine-2′,3′-cyclic monophosphate	1.00	1.0000	**1.31**	0.0413
Pyrimidine, Thymine containing	5,6-dihydrothymine	1.15	0.4695	**1.69**	0.0209
**Cofactors and Vitamins**					
Ascorbate and Aldarate	ascorbate (Vitamin C)	0.95	0.8606	**0.46**	0.0130
Tocopherol	gamma-CEHC	0.62	0.6196	**0.40**	0.0248
Tetrahydrobiopterin	biopterin	1.06	0.8275	**0.21**	0.0444
Benzoate	3-hydroxybenzoate	**2.96**	0.0112	0.51	0.3146
	catechol sulfate	0.48	0.3614	**0.22**	0.0376
	3-(2-hydroxyphenyl)propionate	**2.99**	0.0005	**2.74**	0.0059
Xanthine	1-methylurate	**0.73**	0.0151	0.46	0.5649
	7-methylurate	**0.94**	0.0275	0.27	0.1830
	1,3-dimethylurate	1.07	0.6615	**0.72**	0.0112
	1-methylxanthine	**1.90**	0.0466	1.12	0.9862
	7-methylxanthine	**2.25**	0.0202	0.31	0.1809
Other Phytochemicals	piperidine	0.48	0.3284	**2.59**	0.0176
	2-piperidinone	**0.71**	0.0190	1.16	0.8974
	(15:0)-anacardic acid	1.65	0.8377	**9.38**	0.0448
	apigenin	**21.86**	0.0001	0.46	0.5761
	luteolin	**25.1**	0.0299	0.35	0.7539
	abscisate	**0.31**	0.0138	0.62	0.8696
	enterolactone	**2.00**	0.0278	**2.81**	0.0017
	indolin-2-one	**0.80**	0.0013	1.13	0.6222
	sitostanol	**0.59**	0.0060	**0.79**	0.0767
	Diphenhydramine (drug)	**0.62**	0.0429	1.00	1.0000
	loperamide	**0.65**	0.0324	1.00	1.0000
	salicylate	**1.83**	0.0179	1.08	0.3910
	*N*-methylpipecolate	0.83	0.3601	**1.57**	0.0038

Median scaled relative abundances were assessed for changes at day 28 when compared to baseline (day 0). Bolded fold change values were statistically significant (*p* value > 0.05). * Indicates compounds that did not have a standard, and identification was matched to a database.

**Table 3:** Stool metabolites with statistically significant fold differences in abundance were identified between groups at day 28 following completion of the navy beans or control dietary intervention.

Metabolic Pathway	Metabolite	HMDB	Navy Bean day 28	Control day 28	Fold Difference (NB/Control)	*p*-Value
**Amino Acid**						
Histidine	formiminoglutamate		0.564	5.328	0.11	0.019
	hydantoin-5-propionic acid	HMDB01212	0.829	0.249	3.71	0.016
Lysine	2-aminoadipate	HMDB00510	1.741	0.695	2.69	0.000
	*N*2,*N*6-diacetyllysine		1.553	0.512	3.17	0.032
Leucine, Isoleucine and Valine	4-methyl-2-oxopentanoate	HMDB00695	0.9896	2.5642	0.41	0.024
	*N*-acetylisoleucine		0.722	0.189	0.15	0.039
	3-methyl-2-oxovalerate	HMDB03736	0.856	3.084	0.37	0.034
	3-methyl-2-oxobutyrate	HMDB00019	0.937	2.831	0.41	0.048
Glutathione	5-oxoproline	HMDB00267	1.202	3.316	0.23	0.031
	ophthalmate	HMDB05765	1.480	0.282	3.49	0.031
**Peptide**						
Gamma-glutamyl Amino Acid	gamma-glutamylglutamine	HMDB11738	2.798	1.211	2.60	0.01
**Energy**						
TCA Cycle	malate	HMDB00156	0.690	1.464	0.39	0.029
**Lipid**						
Medium Chain Fatty Acid	caprylate (8:0)	HMDB00482	0.682	4.610	0.1	0.020
	undecanoate (11:0)	HMDB00947	0.853	1.627	0.50	0.015
Polyunsaturated Fatty Acid (*n*3 and *n*6)	eicosapentaenoate (EPA; 20:5*n*3)	HMDB01999	4.014	25.09	0.05	0.031
Fatty Acid (Acyl Glycine)	valerylglycine	HMDB00927	0.775	6.876	0.13	0.048
Monoacylglycerol	1-myristoylglycerol (1-monomyristin)	HMDB11561	0.875	2.246	0.31	0.039
Secondary Bile Acid	glycolithocholate sulfate *	HMDB02639	0.802	1.487	0.39	0.035
	glycocholenate sulfate *		0.171	1.750	0.10	0.026
**Nucleotide**						
Purine	allantoin	HMDB00462	0.173	6.796	0.11	0.049
Pyrimidine	4-ureidobutyrate		0.642	1.921	0.31	0.038
	5,6-dihydrothymine	HMDB1 2308	0.626	0.416	1.56	0.047
**Food Derived**						
Other Phytochemicals	vanillin	HMDB12308	1.228	1.515	0.51	0.036
	nobiletin	HMDB29540	0.528	0.371	1.48	0.048
	salicylate	HMDB01895	2.142	3.094	0.77	0.043

HMDB, Human Metabolome Database; Values presented are fold-difference of the mean relative abundance between navy bean or control group compared to control at day 28. *Indicated compounds that did not have a standard, and identification was match to a database.

## Supplementary Materials

The following are available online at http://www.mdpi.com/2072-6643/11/1/28/s1, Table S1. Navy bean powder food metabolome. Table S2. Metabolites with statistical significance between control diet consumers at 4 weeks compared to baseline. Figure S1: Study design/ consort flow.
