# Supplementary material for: Navy Beans Impact the Stool Metabolome and Metabolic Pathways for Colon Health in Cancer Survivors
Source: Nutrients. 2018 Dec 22;11(1):28. doi: 10.3390/nu11010028 (PMC6356708; doi:10.3390/nu11010028)
Supplement: Supplementary file 1 [file nutrients-11-00028-s001.zip › nutrients-390746-suppls/Supplemental Figure 1 CONSORT.docx]

**Supplemental Figure 1**. CONSORT

Eligibility Criteria:

1. Healthy Adult

2. Minimum of 4 months post colorectal cancer treatment

3. Able to follow a dietary intervention for 28 days

4. No history of food allergies of major dietary restrictions

5. Body Mass Index (BMI) overweight or obese

**Analysis**

**Enrollment**

**Allocation**

Analyzed (n=10)

Stool Metabolome (n=10)

Analyzed (n=10)

Stool Metabolome (n=8)

Control diet (n=10)

Received intervention (n=10)

Withdrawn from study (n=0)

Navy bean diet (n=12)

Received intervention (n=10)

Withdrawn from study (n=2)

Reason for withdrawal

Noncompliant to study protocol (n=1)

Other (n=1)

Randomized

(Sex, BMI and daily caloric intake)

(n=22)

Consort flowchart adapted from Borresen, etal [16].
